# Development and external validation of a transfer learning-based system for the pathological diagnosis of colorectal cancer: a large emulated prospective study

**DOI:** 10.3389/fonc.2024.1365364

**Published:** 2024-04-25

**Authors:** Liuhong Yuan, Henghua Zhou, Xiao Xiao, Xiuqin Zhang, Feier Chen, Lin Liu, Jingjia Liu, Shisan Bao, Kun Tao

**Affiliations:** ^1^ Department of Pathology, Tongji Hospital, School of Medicine, Tongji University, Shanghai, China; ^2^ Department of Pathology, Tongren Hospital, School of Medicine Shanghai Jiaotong University, Shanghai, China; ^3^ Wision Ltd., Chengdu, China; ^4^ Institute of Natural Sciences, MOE-LSC, School of Mathematical Sciences, CMA-Shanghai, SJTU-Yale Joint Center for Biostatistics and Data Science, Shanghai Jiao Tong University, Shanghai, China

**Keywords:** CRC (colorectal cancer), pathological diagnosis, AI-assisted pathological diagnosis, transfer learning, artificial intelligence (AI)

## Abstract

**Background:**

The progress in Colorectal cancer (CRC) screening and management has resulted in an unprecedented caseload for histopathological diagnosis. While artificial intelligence (AI) presents a potential solution, the predominant emphasis on slide-level aggregation performance without thorough verification of cancer in each location, impedes both explainability and transparency. Effectively addressing these challenges is crucial to ensuring the reliability and efficacy of AI in histology applications.

**Method:**

In this study, we created an innovative AI algorithm using transfer learning from a polyp segmentation model in endoscopy. The algorithm precisely localized CRC targets within 0.25 mm² grids from whole slide imaging (WSI). We assessed the CRC detection capabilities at this fine granularity and examined the influence of AI on the diagnostic behavior of pathologists. The evaluation utilized an extensive dataset comprising 858 consecutive patient cases with 1418 WSIs obtained from an external center.

**Results:**

Our results underscore a notable sensitivity of 90.25% and specificity of 96.60% at the grid level, accompanied by a commendable area under the curve (AUC) of 0.962. This translates to an impressive 99.39% sensitivity at the slide level, coupled with a negative likelihood ratio of <0.01, signifying the dependability of the AI system to preclude diagnostic considerations. The positive likelihood ratio of 26.54, surpassing 10 at the grid level, underscores the imperative for meticulous scrutiny of any AI-generated highlights. Consequently, all four participating pathologists demonstrated statistically significant diagnostic improvements with AI assistance.

**Conclusion:**

Our transfer learning approach has successfully yielded an algorithm that can be validated for CRC histological localizations in whole slide imaging. The outcome advocates for the integration of the AI system into histopathological diagnosis, serving either as a diagnostic exclusion application or a computer-aided detection (CADe) tool. This integration has the potential to alleviate the workload of pathologists and ultimately benefit patients.

## Introduction

Colorectal cancer (CRC) ranks as the second-most prevalent global cancer, impacting both men and women (1–3). In recent years, advancements in colonoscopy techniques have significantly improved the identification of precancerous lesions and early-stage cancers (4–7). Notably, artificial intelligence (AI) in computer-aided detection (CADe) for colon polyps, is firmly substantiated by randomized controlled trials and clinical guidelines (8–14). The heightened capability underscores the critical need for precise histopathological diagnoses due to the substantial caseload.

However, AI to enhance histopathological diagnosis for colorectal samples has lagged, experiencing limited progress. Studies indicate that these systems can classify tumors on a slide level with results comparable or superior to traditional observation methods, but primarily in retrospective slide-level analyses and on non-consecutive patient datasets (15–18). This underscores the need for further development and validation of AI technologies in histopathology to ensure their effectiveness in real-world clinical scenarios.

Furthermore, the apparent stagnation of AI in histopathology diagnosis, not only for CRC but in a broad context, can be attributed to a lack of explainability and transparency, stemming from the universal adoption of a two-tiered methodology in analyzing whole slide imaging (WSI): the feature extractor and the aggregation module (19, 20). The feature extractor relies on a convolutional neural network (CNN), processing multiple small tissue regions known as tiles or patches. The aggregation module employs various techniques, from simple max or average pooling to more complex deep learning networks, generating binary predictions for the entire slide or patient (19, 20). However, most validations to date have concentrated on primary performance metrics limited to slide-level binary predictions (21–23). This emphasis on aggregations has overshadowed the evaluation of real image analysis, particularly in recognizing the targeted cancers, i.e., the feature extractors.

While employing aggregation approaches, previous studies have endeavored to localize cancer within WSI. The provision of heatmaps may provide an orientation of highly suspected areas, but their quantitative evaluation remains challenging. Yu et al. conducted internal validations on selected patches of WSI to identify better feature extractors (14). Griem et al. undertook exhaustive pixel-level cancer segmentation on 30 slides, achieving a Dice score of 0.895 (24). However, the complexity and annotation workload associated with this approach hinders its scalability to large datasets. Thus, a crucial need in the field is a practical, measurable method to assess AI’s proficiency in recognizing and localizing cancer features within slides, aiming to improve prediction transparency and explainability.

**Figure 1:**
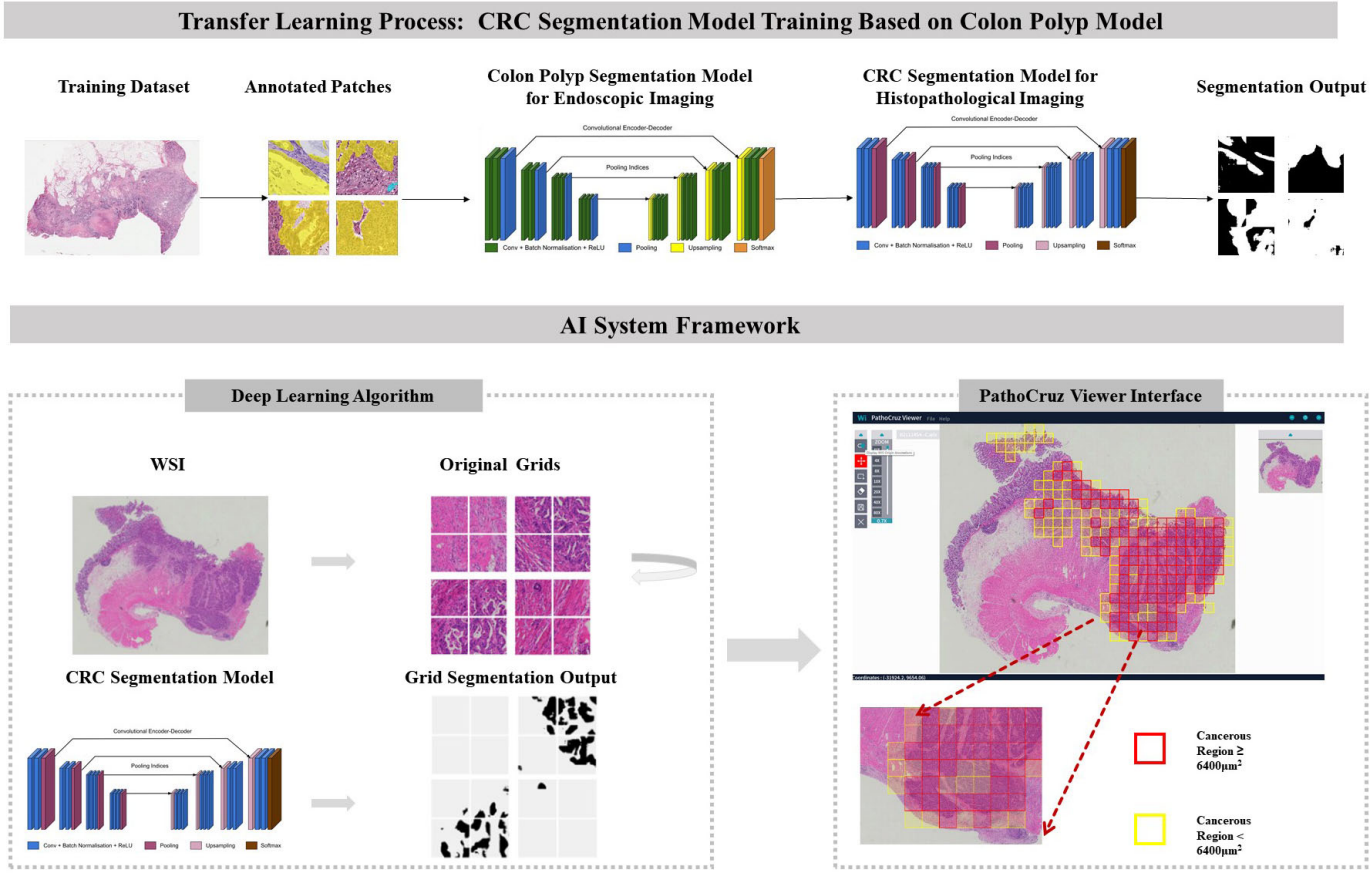
Transfer Learning Model Framework. CRC Segmentation Model Training Process: The CRC segmentation model was derived through transfer learning from a pre-existing colon polyp segmentation model. This transfer learning process involved utilizing histological patches with pixel-level annotations extracted from whole slide imaging sourced from The Cancer Genome Atlas (TCGA) database. AI System Workflow: The AI system processes the whole slide images, segments it into grids, and predicts the presence or absence of cancerous tissue. The viewer interface displays marked regions with bounding boxes indicating detected cancerous tissue, based on segmentation results. Final AI predictions for individual grids and the entire slide are shown, with red or yellow bounding boxes indicating cancerous regions ≥ or < 6400 μm², respectively. The color of the bounding boxes was disregarded in this study. WSI, Whole slide image; CRC, Colorectal cancer.

In this context, we developed an AI system focused on detecting histological features of CRC and reporting imaging recognition results per location. Exhaustive validations were conducted at the sub-slide level, working at a granularity of 0.5*0.5 mm² grid segments within the fully meshed WSI (**Figure 1**). This approach minimizes ambiguity by directly translating AI’s identification of cancerous regions into the slide-level output without adopting any aggregation models. This process involved transferred learning from a proven successful segmentation model originally designed for gastrointestinal endoscopy (25).

This study represents a pioneering effort in the field of histopathological deep-learning research. The evaluation of performance metrics and the observation of pathologist behavioral changes at this fine-grained level provided unprecedented insights into the reliability of AI in localizing cancerous regions and how on-slide markers influenced pathologists’ decisions. We anticipate that our findings will offer valuable insights, potentially catalyzing a revolutionary transformation in the development of AI applications in CRC and other histopathological diagnosis areas.

## Method

Before the external validation through performance assessments and a simulated prospective reader study, the deep learning algorithm had been finalized and incorporated into the software system outside the institution.

### Algorithm development

In the context of prior work focused on colorectal polyp detection in endoscopy imaging, the customized SegNet model has demonstrated proficiency with a local-feature-oriented recognition capability through rigorous testing (6, 25, 26), including randomized controlled trials (RCTs) (6, 8, 10, 11, 27). Therefore, rather than training an entirely blank network, a transfer learning approach was adopted with the polyp segmentation model under white-light endoscopy as the start point (28). This approach is particularly crucial in histopathological feature recognition, where local features play a significant role in the absence of prominent macro features (29). The new algorithm commenced its training process with an initial set of 15,279,170 parameters. This training utilized an extensive dataset comprising 431,078 pixel-level annotated patches extracted from 219 WSIs sourced from The Cancer Genome Atlas database (TCGA, https://www.cancer.gov/ccg/research/genome-sequencing/tcga).

The algorithm employed a fully-supervised, multilayered convolutional neural network (CNN) based on the SegNet system. The SegNet architecture comprised an encoder and a decoder, facilitating the capture and computation of annotated morphological features for each input image (30). In sequence, the images underwent warping into a binary mask through a probability calculation. In this binary mask, a value of 1 denoted the presence of cancer, while 0 indicated the absence of cancer, aligning with the intensity of the pixels. The AI system generates predictions based on 0.25*0.25 mm^2^ grids but presents the results on 0.5*0.5 mm^2^ grids for improved human visual comfort. Additionally, the 0.5*0.5 mm^2^ grids are better suited for pathologists to capture gland structures for labeling. It is important to note that the visualization of the 0.5*0.5 mm^2^ grids is derived from the prediction results of the 0.25*0.25 mm^2^ grids. Specifically, a positive label is assigned to any 0.25*0.25 mm^2^ grid where the cumulative area of positive predictions exceeds 625 µm²; otherwise, it is labeled as negative.

The development dataset and internal validation results are described in **Supplementary Material**.

### AI software system description

The algorithm was encapsulated in the PathoCruz software system (Wision Ltd., Shanghai, China). The AI system automatically meshes the original WSI into 0.5*0.5 mm^2^ square grids according to the Micron Per Pixel (MPP) of the WSI (**Figure 1**). Annotating the bounding boxes of each grid offers the full breadth localization of cancerous tissues on visualized slides. The AI system provides a WSI Viewer to enable pathologists to switch from the original scanned slide image to the full-meshed grid view with AI labels. Images can be zoomed in to examine and measure targets at the viewer interface.

The model and software have remained unmodified since established. The development and validation process are summarized in **Supplementary Material**.

### Study design

We conducted the standalone performance validation as well as an emulated prospective study, to assess the diagnostic behavior impact of employing the AI system for grid-level CRC detection in histopathological diagnosis.

Four pathologists participated in this emulated prospective study: three junior pathologists (JP) with less than 5 years of experience and one senior pathologist (SP) with 5-10 years of experience. Their involvement included conducting diagnostic reviews and annotations on WSI grids of colorectal specimens obtained from consecutive patients.

**Figure 2:**
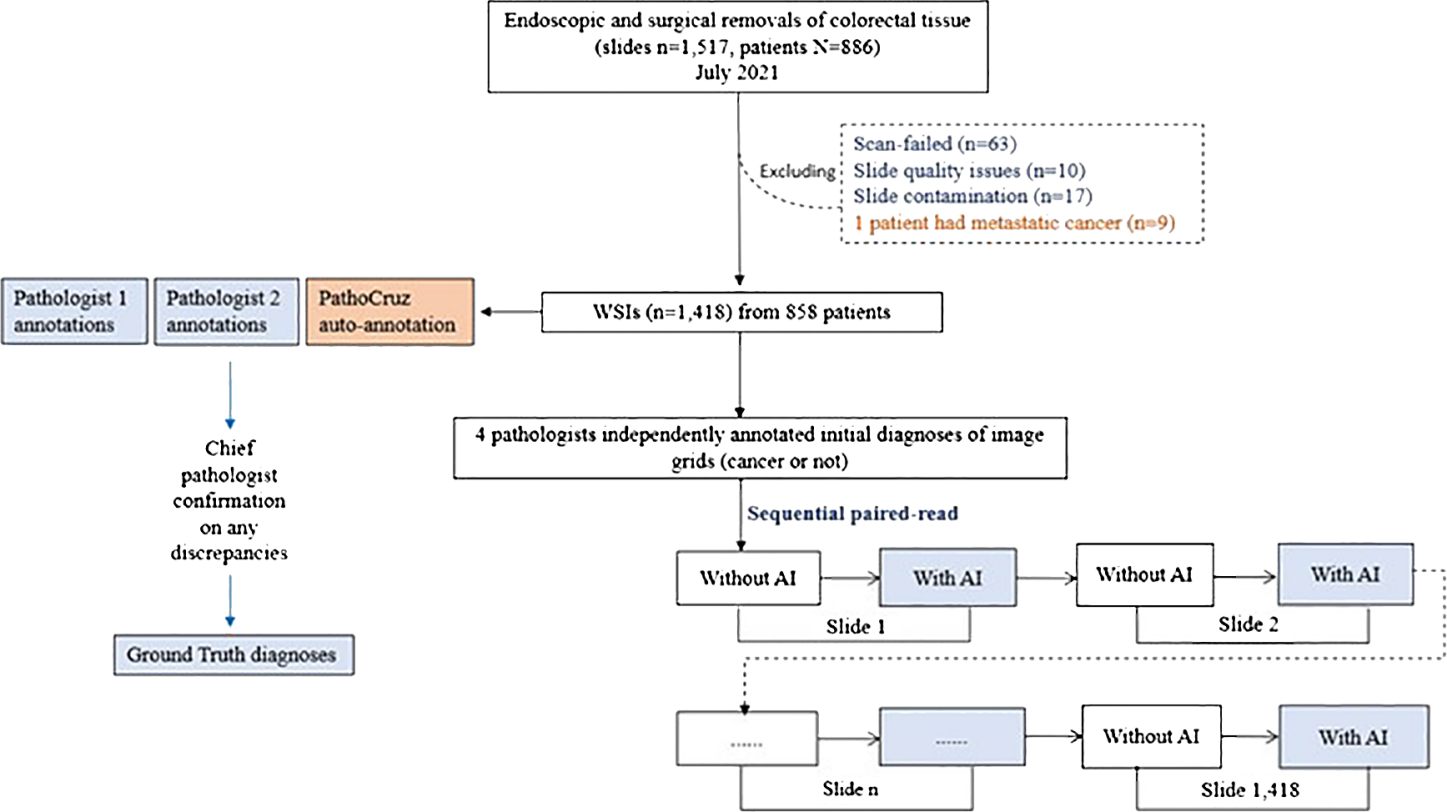
Study Flowchart. Ground truth acquisition and the diagnostic study are distinct and conducted independently.

The WSI dataset was digitized with slides from consecutive patients between July 1, 2021, and July 31, 2021. This time frame covers 12 months preceding the commencement of our study, ensuring an adequate washout period for the participating pathologists. Patients were eligible for inclusion in the study if they had undergone endoscopic and surgical procedures on the colon or rectum, with tissue samples sent for histopathological assessment. Exclusion criteria were applied to patients with metastatic colon cancer originating from other organs. Additionally, slides were excluded if they experienced scan failures, exhibited quality issues, or showed signs of contamination. The emulated prospective study procedure is described in **Supplementary Material** (**Figure 2**).

### External data collection

A total of 1,517 slides from 886 consecutive patients who underwent endoscopic or surgical removal from the colon or rectum in July 2021 were retrieved from the Department of Pathology at Shanghai Tongren Hospital, China. A total of 1418 slides from 858 patients were finally included by the inclusion and exclusion criteria. The colorectal specimens were from either endoscopic or surgical removal and processed with the hematoxylin-eosin (H&E)-stained formalin-fixed paraffin-embedded (FFPE) method. All slides were digitized at 40x magnification using a Digital Scan System SQS-1000 (TEKSQRAY, China) from June 12 to June 30, 2022.

### Ground truth

Ground truth revolves around determining the presence of cancer within each image grid. Each WSI was subdivided in a full mesh style into disjoint grids, each measuring 0.5*0.5 mm^2^. The slide-level ground truth, on the other hand, was established by assessing whether any of the grids within a WSI contained cancer (31).

The process of establishing the ground truth was conducted before the study procedure, which was separate from the below diagnostic study procedure. Two pathologists, each with between 5 and 10 years of experience, were tasked with independently annotating grids that exhibited signs of cancer presence on each WSI. Subsequently, a dedicated software program automatically compared their annotations with the outputs generated by the AI system. If consensus was reached among two pathologists and AI, then the ground truth of grids was made. Any grids that exhibited disagreements between the two pathologists or discrepancies between either pathologist and the AI output were escalated to the chief pathologist for a final decision, and the ground truth of these grids was made by the chief pathologist.

It’s important to note that the two pathologists conducting the annotations were blind to the AI outputs, and they did not participate as WSI readers in the study. Similarly, the chief pathologist remained blinded to both the AI outputs and the annotations made by the two pathologists.

### Outcome measures

For the justification of the AI facilitation on pathological diagnosis of CRC, we pre-defined the performance metrics as the sensitivity and specificity of the four pathologists and their averages, with the aid of AI versus without, respectively at the grid and slide levels. In addition, the AI standalone performance was evaluated by yielding the receiver operating characteristic (ROC) curve following the trapezoidal rule (32). Furthermore, modifications with AI assistance, false negative(FN) to true positive (TP), TP to FN, true negative (TN) to false positive (FP), and FP to TN were analyzed.

### Statistical analysis

The primary analysis was composed of per-grid and per-slide sensitivity/specificity, compared with the ground truth. The results were analyzed by calculating 2-sided 95% confidence intervals (CIs) of variance estimate, using the Wald Interval model (R version 4.2.0). The P value was calculated using McNamara’s test.

The WSI was divided into 0.5*0.5 mm² disjoint grids. AI labeling a grid with cancerous tissue constituted a true positive (TP), while the absence of a label on such a grid was deemed a false negative (FN). Sensitivity at the grid level was defined as TP/(TP+FN). For grids without cancerous tissue, the absence of a label indicated a true negative (TN), whereas a label marked a false positive (FP). Specificity at the grid level was calculated as TN/(TN+FP).

At the slide level, metrics were directly derived from grid-level results. A positive slide required at least one TP grid to call TP, otherwise it was an FN slide. For negative slides, the absence of FP grids determined true negatives (TN). Sensitivity and specificity were calculated accordingly.

## Results

### Study population

A total of 1517 WSIs of colorectal specimens from 886 patients who underwent endoscopy and/or surgical resection were consecutively enrolled, of these, 858 patients with 1,418 WSIs were included. Among them, 238 slides were obtained from 23 patients who underwent surgery, and 1,180 slides were from 847 patients who underwent endoscopy. Notably, 12 patients had both procedures.

The patients’ ages for endoscopic procedures ranged from 18 to 90 years old with a mean age of 58.51( ± 12.31) years and were male 57.11%. 42 patients were diagnosed with colorectal carcinoma (including carcinoma in situ) in at least one slide, while 816 patients were diagnosed as not having CRC (**Table 1** **).**

**Table 1:** Patient demography.

Patient demography			
	Characteristics	Patients for Endoscopic Procedures (n=847)	Patients for Surgical Procedures (n=23)
**Sex**	Female, n (%)	362 (42.74)	11 (47.82)
	Male, n (%)	485 (57.26)	12 (52.17)
**Patient age (yrs)**	Mean (±SD)	58.37 (±12.29)	69.48 (±9.00)
	Median	60	68
	Min–max	18-90	53-86
**Age category**	10-39, n (%)	75 (8.85)	0 (0)
	40-69, n (%)	637 (75.21)	14 (60.87)
	70-99, n (%)	135 (15.93)	9 (39.13)
**Location**	Left colon n (%)	445 (52.54)	17 (73.91)
	Right colon, n (%)	160 (18.89)	6 (26.08)
	Left & right colon, n (%)	242 (28.57)	0 (0)
**Tumor Size (cm)**	< 5 cm, n (%)	NA	9 (39.13)
	≥ 5 cm, n (%)	NA	14 (60.87)
**Adenoma**	conventional colorectal adenoma, n (%)	451 (53.25)	NA
	serrated adenoma, n (%)	12 (1.41)	NA
	hyperplastic polyp, n (%)	324 (38.26)	NA
	inflammatory polyp, n (%)	41 (4.84)	NA
	rare types (e.g. hamartoma), n (%)	5 (0.59)	NA
**Adenocarcinoma**	adenocarcinoma NOS, n (%)	6 (0.71)	14 (60.87)
	adenoma-like adenocarcinoma, n (%)	3 (0.35)	7 (30.43)
	micropapillary adenocarcinoma, n (%)	0	1 (4.35)
	mucinous adenocarcinoma, n (%)	0	1 (4.35)
	unknown, n (%)	5 (0.59)	0 (0)
**Low-grade dysplasia**	conventional colorectal adenoma, n (%)	434 (51.24)	NA
	serrated adenoma, n (%)	12 (1.42)	NA
**High-grade dysplasia**	conventional colorectal adenoma, n (%)	17 (2.01)	NA
**Cancerous**	cancerous, n (%)	14 (1.65)	NA
**No-dysplasia**	no-dysplasia, n (%)	370 (43.68)	NA
**Differentiation**	well, n (%)	NA	1 (4.35)
	moderate, n (%)	NA	16 (69.56)
	poor, n (%)	NA	6 (26.09)
**Lymph node metastasis**	N0, n (%)	NA	15 (65.22)
	N1, n (%)	NA	6 (26.09)
	N2, n (%)	NA	2 (8.70)
**Invasion depth**	T1, n (%)	NA	3 (13.04)
	T2, n (%)	NA	2 (8.70)
	T3, n (%)	NA	11 (47.83)
	T4, n (%)	NA	7 (30.43)
**Distant metastasis**	M0, n (%)	NA	21 (91.30)
	M1, n (%)	NA	2 (8.70)
**TNM**	I, n (%)	NA	5 (21.74)
	II, n (%)	NA	8 (34.78)
	III, n (%)	NA	8 (34.78)
	IV, n (%)	NA	2 (8.70)

NA, not applicable.

### AI standalone performance

For the grid-level evaluation, among a total number of 3,589,476 grid images, 3,513,562 grids with benign-only features were confirmed, and there were 75,914 grid images with cancer presence. Overall, AI demonstrated robust and outstanding detection power for the grid-level evaluation, with a per-grid sensitivity of 90.25%; (95%CI, 90.04%, 90.46%), and a per-grid specificity of 96.60%; (95%CI, 96.58%, 96.62%), with an overall positive likelihood ratio (LR) of 26.54, and a negative LR of 0.101 (**Table 2A**).

**Table 2:** AI standalone performance at the slide and grid levels.

A: Grid-level performance of AI										
Whole slide images		No. of grids	TP	FN	TN	FP	Sensitivity	Specificity	Positive likelihood ratio	Negative likelihood ratio
**All sample**	Cancer or HGD	297,403	68,510	7,404	211,823	9,666	90.25%; 95%CI (90.04%, 90.46%)	96.60%; 95%CI (96.58%, 96.62%)	26.54	0.101
	Benign	3,292,073	NA	NA	3,182,282	109,791	NA			
**Endoscopic Removals**	Cancer or HGD	103,355	19,961	2,201	75,028	6,165	90.07%; 95%CI (89.67%, 90.46%)	96.49%; 95%CI (96.47%, 96.51%)	25.66	0.103
	Benign	3,188,512	NA	NA	3,079,799	108,713	NA			
**Surgical Resections**	Cancer or HGD	194,048	48,549	5,203	136,795	3,501	90.32%; 95%CI (90.07%, 90.57%)	98.12%; 95%CI (98.06%, 98.18%)	48.04	0.099
	Benign	103,561	NA	NA	102,483	1,078	NA			
B: Slide-level performance of AI										
Method of sampling		Total No. WSI	TP	FN	TN	FP	Sensitivity	Specificity	Positive likelihood ratio	Negative likelihood ratio
**Overall**		1,418	163	1	856	398	99.39%; 95%CI (98.20%, 100.58%)	68.26%; 95%CI (65.69%, 70.84%)	3.13	0.009
**Endoscopic Removals**		1,180	36	0	776	368	100.00%; 95%CI (100.00%, 100.00%)	67.83%; 95%CI (65.13%, 70.54%)	3.11	0
**Surgical Resections**		238	127	1	80	30	99.22%; 95%CI (97.69%, 100.74%)	72.73%; 95%CI (64.40%, 81.05%)	3.64	0.011

HGD, high-grade dysplasia; NA, not applicable.

For the ground truth diagnosis at the slide level, 164 WSIs were positive (malignant tissue confirmed), and 1254 slides were negative (benign). AI exhibited an impressive stand-alone per-slide sensitivity of 99.39% (95% CI, 98.20%-100.00%) and a per-slide specificity of 68.26% (95%CI, 65.69%-70.84%) in the current cohort, with an AUC of 0.966, in which for the endoscopic negative LR of 0.009 (**Table 2B**).

On surgical resection slides, AI achieved sensitivity 99.22% (127/128) and specificity 72.73% (80/110), while on endoscopic resection slides with sensitivity 100% (36/36) and specificity 67.83% (776/1144). Besides, the AI’s diagnostic results of 12 patients who underwent both endoscopic and surgical resection are consistent across both diagnoses.

### AI impact on pathologists’ diagnosis

**Figure 3:**
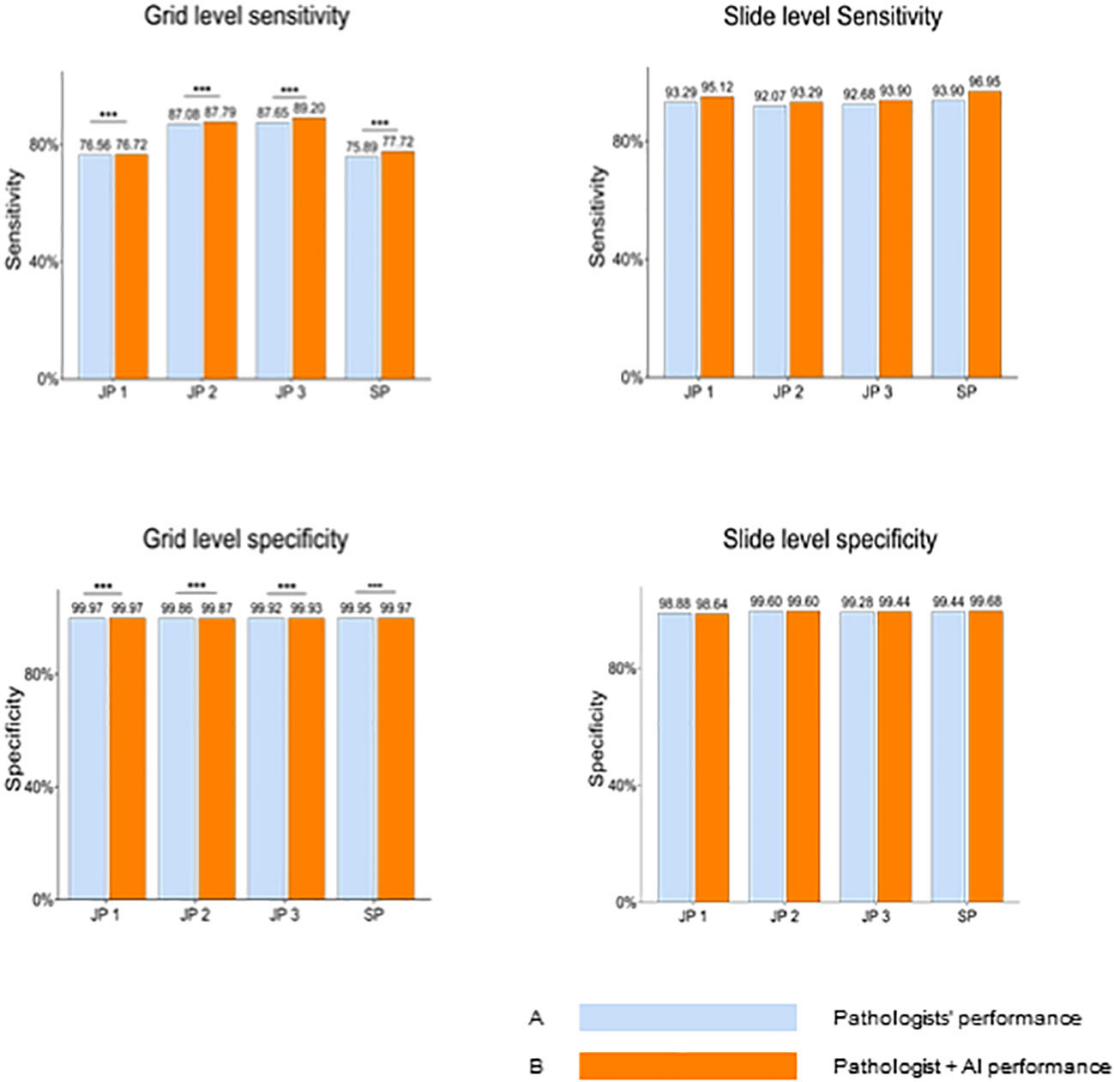
Pathologists’ overall performance without AI Versus with AI at the gird and slide levels. Blue bars represent pathologists' performance without AI assistance, while orange bars indicate performance with AI assistance. At the grid level, pathologists demonstrate significantly enhanced sensitivity and specificity when aided by AI. JP, Junior Pathologist; SP, Senior Pathologist.

The performance differences between the 4 pathologists between the two groups, assisted or unassisted with AI, are shown in **Figure 3**. Pathologists with AI demonstrated higher sensitivity and specificity compared with corresponding initial diagnoses, at the grid level, with the sensitivity rising from 81.80% (95% CI; 81.52%, 82.07%) to 82.86% (95% CI; 82.59%, 83.13%). The average per-grid specificity of 4 pathologists also increased significantly while assisted by AI, from 99.93% (95% CI; 99.92%,99.93%) to 99.94% (95%CI; 99.93%, 99.94%). All Pathologists also presented a positive trend in slide-level diagnosis accuracy.

**Figure 4:**
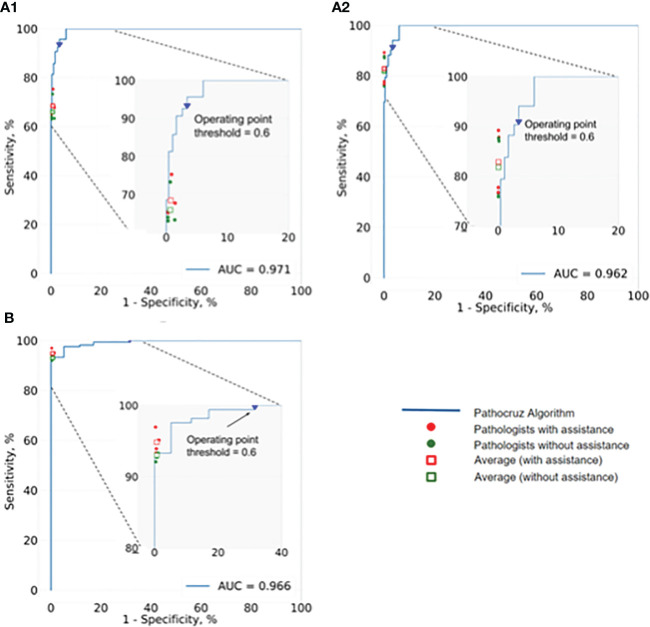
ROC of AI system and the performance change of 4 pathologists with and without the aid of the AI. **(A1)** Analysis of the positive WSIs with cancerous area ≤50 mm^2^ at the grid level. AI achieved AUC=0.971, and on average pathologists’ sensitivity gained from 64.43% (95% CI: 63.62%, 65.24%) to 66.99% (95% CI: 66.19%, 67.79%), whereas there was no significant difference in specificity. **(A2)** Analysis of all positive WSIs at the grid level. The AUC of AI was 0.962. All pathologists improved significantly with the AI aid, on average, increasing from 81.80% (95% CI: 81.52%, 82.07%) to 82.86% (95% CI: 82.59%, 83.13%) for sensitivity, and 99.93% (95% CI; 99.92%, 99.93%) to 99.94% (95% CI; 99.93%, 99.94%) for specificity. **(B)** Analysis of all positive WSIs at the slide level. Pathocruz achieved AUC=0.966 among all-slide detection, and the accuracy of each pathologist was gained with Pathocruz assistance, without statistically significant change, 92.99 % (95% CI; 89.08%, 96.90%) to 94.82 % (95% CI; 91.42%, 98.21%) on average, and 99.30 % (98.84%, 99.76%) to 99.34 % (95% CI; 98.89%, 99.79%) respectively.

Next, we investigated the performance of pathologists aided by AI at size ≤ 50mm^2^ cancerous regions, as well as overall performance. AI demonstrated an AUC of 0.966 at the slide level of cancer detection. The size of the cancerous area contained in all WSIs ranging from 0.5mm^2^(minimum) to 349.25mm^2^ (maximum). For these positive slides that contained cancerous regions size ≤ 50mm^2^, AI achieved a high AUC of 0.971 at the grid level and 0.962 for all WSIs. We also integrated the performance of the 4 pathologists with and without AI aid, into the corresponding receiver operating characteristic (ROC) curves (**Figure 4** **),** AI demonstrated higher detection performance than any pathologist at the operation threshold, and all pathologists made improvements while aided by AI, especially examine the small-sized (≤ 50mm^2^) cancerous areas that are easily overlooked.

**Figure 5:**
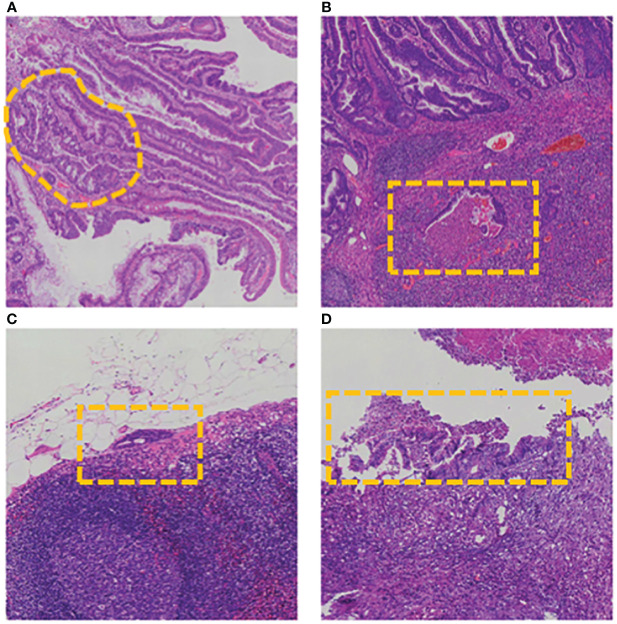
Examples of Slide-Level Diagnostic Corrections Aided by AI Output on Grids. Image illustrates cases where there are diagnostic changes at the slide level, specifically due to grid-level assistance by AI (FN to TP). The areas circled by yellow marks are typical cancerous tissues. **(A)** Tubular structures. **(B)** Cancer tissue breaking through the mucosal muscular layer. **(C)** Small areas of epithelial tissue migrating into lymphatics. **(D)** Epithelial tissue migrating into lymphatics. FN, false negative; TP, true positive.

Pathologists’ decision change was quantified by analyzing the pathologists’ revision of their initial diagnosis at the grid level. **Table 3** shows that the four pathologists have collectively revised 3,169 grids from FN to TP,1,596 grids from FP to TN, 574 grids from TN to FP, and 158 grids from TP to FN. We discovered that mostly the positive influence AI had on readers significantly outnumbered the negative influence. **Figure 5** displays grids showcasing pathologist diagnostic changes at the slide level assisted by AI (from FN to TP).

**Table 3:** Pathologists’ revision at the grid level while being exposed to AI results.

Pathologists	Revision on positive grids		p-value*	Revision on negative grids		p-value*
	(3,327 of 75,914)			(2,170 of 3,513,562)		
	FN to TP	TP to FN		FP to TN	TN to FP	
	(n=3,169)	(n=158)		(n=1,596)	(n=574)	
JP 1	122 (98.39%)	2 (1.61%)	<0.0001	15 (22.73%)	51 (77.27%)	<0.0001
JP 2	540 (99.08%)	5 (0.92%)	<0.0001	536 (82.08%)	117 (17.92%)	<0.0001
JP 3	1,321 (90.23%)	143 (9.77%)	<0.0001	823 (67.46%)	397 (32.54%)	<0.0001
SP	1,403 (99.29%)	10 (0.71%)	<0.0001	594 (91.10%)	58 (8.90%)	<0.0001

*p-value from two proportions McNemar's-test.

TP, True positive; FN, false negative; TN, True negative; FP, false positive.

JP, Junior Pathologist.

SP, Senior pathologist.

There are overlapped grids revised by pathologists.

**Figure 6:**
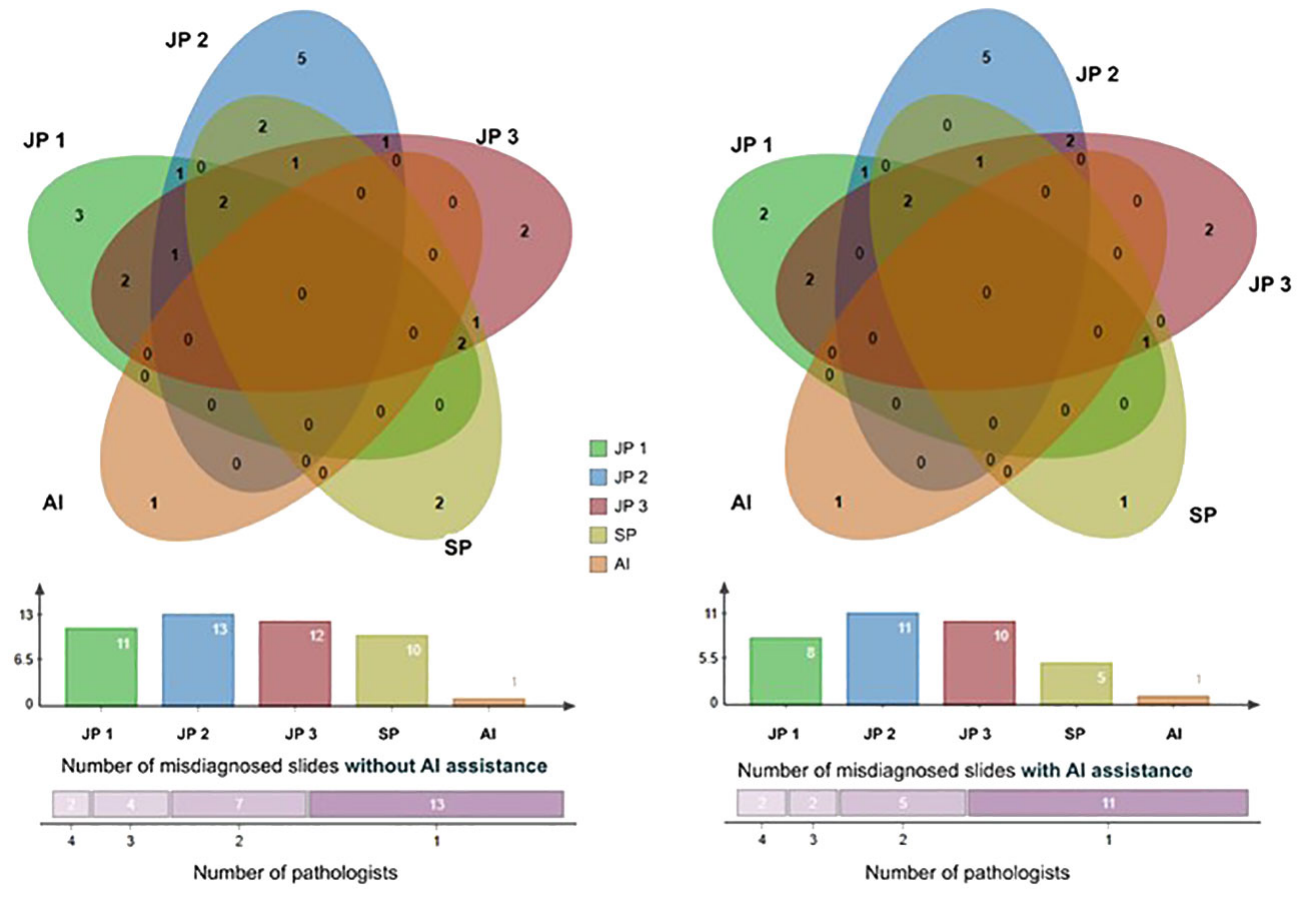
The Venn diagram comprises five circles representing the number of missed cases by the four pathologists and the AI system. Overlapping areas indicate cases missed by more than one pathologist. The Venn graph illustrates the relationships between misdiagnosed cases by each pathologist alone (left) versus with AI assistance (right); The Column charts show the number of cases each pathologist or AI missed. There were 2 slides misdiagnosed by 4 pathologists, 4 slides misdiagnosed by 3 pathologists, 7 by 2, and 13 by 1 initially, and this number of misdiagnosed slides was reduced with the aid of AI. JP, Junior Pathologist; SP, Senior Pathologist.

We utilized a Venn diagram to further analyze instances of misdiagnoses by physicians, with and without the aid of AI, as well as cases where both AI and physicians were incorrectly (33)(**Figure 6**). A “missed slide” was defined as a slide where all positive grids were overlooked by the observer. Among the 1,418 Whole Slide Images (WSIs) examined, we identified 26 slides that were missed either by pathologists or AI. Of these cases, 25 were overlooked by pathologists, while AI missed one. Notably, none of the slides were missed by both pathologists and AI based on their initial independent diagnoses. Additionally, when utilizing the AI tool, the four pathologists revised their diagnosis for a total of 12 WSIs. For each individual pathologist, the corrections involved 3 WSIs, including 2 from surgical resections, 2 exclusively from endoscopic procedures, 2 with 1 from each procedure, and 5 with 4 originating from endoscopy. This underscores the collaborative potential of combining human expertise with AI assistance to enhance diagnostic accuracy.

## Discussion

The novelty of our study encompasses three key aspects: successful transfer learning from a proven local feature-oriented model, the groundbreaking decision to forego aggregation, allowing for location-level validation, and an emulated prospective study design with large consecutive patient data.

Firstly, transfer learning has been proven effective in medical imaging (34, 35), and our study pioneers the application of a segmentation model from endoscopy to histopathology. The SegNet model was initially trained for local features, such as pit patterns and microvessels on polyps for colonoscopy video frames (20). Through engineering efforts and 2,338,000 epochs, the focus was shifted towards identifying distinctive characteristics such as substantial loss of cell polarity, markedly enlarged nuclei with prominent nucleoli, a dispersed chromatin pattern, and atypical mitotic figures. This engineering principle aligns with the WHO histopathological criteria for CRC, ensuring adherence to established standards (36).

Secondly, without adopting any slide-level aggregation model, our AI’s outputs remain explainable. This allows for concentrated validation of the algorithm’s image recognition capabilities, forming the foundation of trustworthiness for clinical adoption. The grid-level prediction of malignancy, represents a groundbreaking technological advancement in the field of AI-powered histopathology, providing a thorough view of the full breadth localization of cancer precisely at the granularity of 0.5 x 0.5 mm² grid segments, and collectively depicting the extent of cancer across the entire slide. The system exhibits high prediction stability with an area under the curve (AUC) of 0.962 at the grid level. Inherent to this, at the slide level, only one slide of adenoma-like adenocarcinoma is missed by the AI, and 68.26% of benign slides show no false alarms on any grid. The algorithm’s high discriminating power, consistency, and generalizability have been validated across patient age, sex, tissue resources, and cancer subtypes.

The evaluation of our AI system’s performance is predicated on consecutive patients’ data rather than a curated dataset, rendering the assessment of positive and negative likelihood ratios (LRs) of paramount importance for its clinical utility (37). The negative LR was determined to be less than 0.01 at the grid level, and approximately 0.1 at the slide level, signifying that AI can furnish highly reliable results for identifying benign regions and slides. This discovery paves the way for the potential application of AI to exclude CRC diagnosis in the clinical workflow. In terms of positive LRs, an LR greater than 3 at the slide level implies the possibility of some false positives when employing AI for slide screening for malignancies. Notably, at the grid level, a positive LR exceeding 10 strongly suggests that any highlighted grids warrant a meticulous examination. Consequently, this AI system can be effectively utilized both as a diagnosis exclusion tool before the diagnostic review and as a CADe tool during the review, enhancing CRC diagnostic accuracy and decision-making capabilities of pathologists.

Most importantly, the paramount contribution of the current study lies in the exploration, within an emulated prospective setting, of the impact of AI assistance on pathologists. Our results unequivocally show that, with AI assistance, pathologists significantly improved their performance of histopathological diagnosis at the grid level (P<0.001), specifically resulting in increased true positives and more true negatives. Moreover, the combined clinician-AI diagnosis lowered the rate of incorrectly diagnosed slides. Notably, the great credibility of the study results attributed to rigorous design, for example, to minimize the potential bias, consecutive patients that relatively represent the desired target population were included, and the performance metrics were pre-defined before the initial of the research. Our deep learning evaluation methodology aligned with the Level IV study principles that were advised by Kleppe et al. in their recent review (38).

We compared the performance change of the four pathologists when utilizing AI versus their unassisted reads at the grid and slide level. With the assistance, significant maximized sensitivity was consistently achieved at the grid level with associated modest improvement of specificity, in contrast to the pathologists’ initial annotations. Also, there was a positive trend observed in per-slide accuracy, highlighting the substantial performance gains of all the pathologists. Specifically, we have interpreted pathologists’ revision of their initial reads while being exposed to AI outcomes, and the accuracy improvement driven by AI was statistically confirmed on both cancerous (positive) and benign (negative) grid-wise and WSI-wise. The four pathologists have corrected their annotations on a total of 3,169 grids (4.21% of all positive grids) from FN to TP, and on 1,596 grids from FP to TN, although there are also incorrect revisions of girds driven by AI. For example, several 158 grids were revised from TP to FN, and they presented an insignificant impact on slide-level diagnosis results, as pathologists have made zero revisions from TP to FN for slide-level diagnosis. However, pathologists have become correct after changing their diagnosis on 9 WSIs, from FN to TP, driven by AI. These findings indicate that AI-driven annotations that resulted in accuracy gains significantly outnumbered those that resulted in accuracy loss (P<0.001), and AI could aid pathologists in accurately identifying cancerous regions at a more precise scale, which potentially leads to improved patient-level diagnosis.

Furthermore, we analyzed the 26 false-negative slides, missed by pathologists (25) and AI (1). There was no overlap between the slides missed by AI and the pathologists, which indicates an AI and human complementary mechanism. In detail, the 4 pathologists collectively missed two slides, with a maximum of four slides missed simultaneously by three pathologists and a maximum of seven slides missed simultaneously by two pathologists. Commonly, these slides contained small or isolated cancerous areas, and moderate to poorly differentiated lesions. The AI system missed one slide, which was identified as adenoma-like adenocarcinoma, an uncommon variant of CRC (39). This case shared very similar characteristics with villous adenoma, posing a challenge for differentiation primarily based on cytological features. However, AI is significantly helpful for pathologists in reducing the risk of overlooking cancer, particularly in situations involving small tumors, lymph node metastasis, and omental metastasis. Collectively, the 4 pathologists revised 22 times for slide diagnosis to become correct, and the senior pathologist made the most valid revision while aided by AI, this may also reveal the interobserver variability between pathologists (40). The AI system appears to play a pivotal role in mitigating inaccurate diagnoses by offering objective and consistent results, which carries significant clinical importance.

Our study has several limitations. Firstly, it is a single-center external study, which may limit the generalizability of our findings. To enhance the robustness of testing the AI system, future research should consider incorporating data from multiple sources, scanning devices, and patient populations. Secondly, our reader study procedure deviates from the routine of histological diagnostics, as its primary goal is to measure the standalone performance of the AI and its impact on human readers at the sub-slide level. Thirdly, it is also important to note that our study was not explicitly designed to measure efficiency metrics when assisted by AI, an aspect more appropriately examined in prospective studies. Lastly, consecutive patient inclusion in our study reflects natural distribution, but may not have yielded a satisfactory volume of positive cases. Future studies should consider enriched datasets focusing on positive cases for a more comprehensive assessment.

In conclusion, this research directly evaluates AI performance in CRC recognition at a highly granular level without relying on slide-level aggregation, demonstrating its potential for diagnosis exclusion and CADe applications. Involving pathologists in this study yields promising results, highlighting AI as a valuable complement to human expertise, enhancing diagnostic accuracy. To further explore this synergy, we advocate for a prospective randomized controlled trial. This study, by advancing our understanding of AI and human expertise integration, is poised to significantly improve diagnostic precision, ultimately elevating patient care quality in pathology.

## Data availability statement

The original contributions presented in the study are included in the article/**Supplementary Material**. Further inquiries can be directed to the corresponding author.

## Ethics statement

The studies involving humans were approved by the Human Ethics Committees, Tongren Hospital, Shanghai Jiao Tong University School of Medicine (the approval number is 2022-028-02). It was registered with the Chinese clinical trial registry under the identifier ChiCTR2300076271. The studies were conducted in accordance with the local legislation and institutional requirements. Written informed consent for participation was not required from the participants or the participants’ legal guardians/next of kin in accordance with the national legislation and institutional requirements.

## Author contributions

LY: Data curation, Writing – original draft, Writing – review & editing. HZ: Formal analysis, Writing – original draft. XX: Methodology, Software, Validation, Visualization, Writing – review & editing. XZ: Investigation, Supervision, Validation, Visualization, Writing – original draft. FC: Data curation, Formal analysis, Writing – review & editing. LL: Data curation, Methodology, Writing – review & editing. JL: Investigation, Methodology, Resources, Software, Writing – original draft. SB: Conceptualization, Supervision, Writing – original draft. TK: Conceptualization, Supervision, Validation, Writing – review & editing, Methodology.
