# Supplementary material for: Development and external validation of a transfer learning-based system for the pathological diagnosis of colorectal cancer: a large emulated prospective study
Source: Front Oncol. 2024 Apr 25;14:1365364. doi: 10.3389/fonc.2024.1365364 (PMC11079287; doi:10.3389/fonc.2024.1365364)
Supplement: Supplementary file 1 [file DataSheet_1.docx]

**Supplementary Material**

The supplementary material contain three parts: a complementary figure, a detailed description of the AI model and the study procedure.

**Figure S1. Ground Truth Generation Demonstration**

**
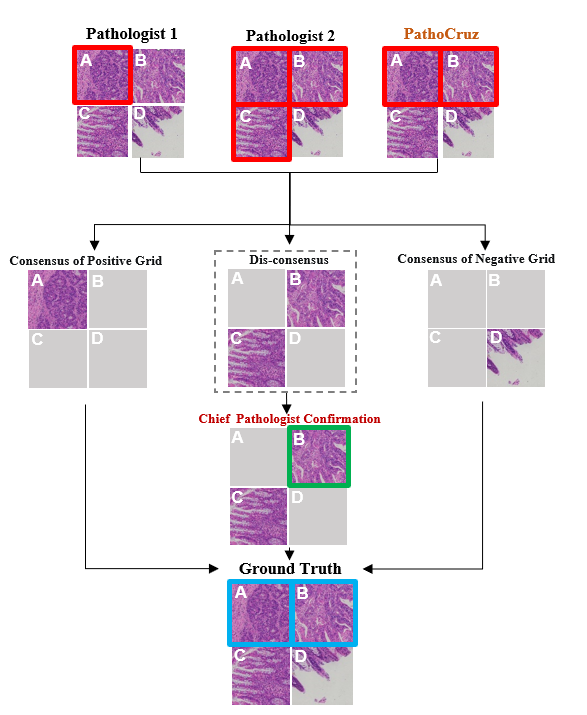
**

**Establishment process for ground truth：**

Two senior pathologists and AI are independently annotating the grids showing signs of cancer on each WSI with red boxes. Subsequently, a dedicated software program automatically compared their annotations with the outputs generated by the AI system. Any grid that exhibits disagreement between the two pathologists or between the pathologist and the AI output (green box) is escalated to the chief pathologist for a final decision. And the grid marked in blue is the final established ground truth.

**Note:** Two pathologists conducting the annotations were kept blind to the AI outputs, and they did not participate as WSI readers in the study. Similarly, the chief pathologist remained blinded to both the AI outputs and the annotations made by the two pathologists.

***Model detailed description***

***Algorithm Development Dataset***

The development dataset comprised Whole Slide Images (WSIs) of colorectal specimens obtained from The Cancer Genome Atlas (TCGA), originating from surgical removals across 18 global institutions. These histopathological slides were prepared using either the H&E stained FFPE method or flash-frozen technique, and scanned the Leica AT-2 WSI scanner (Leica Biosystems, IL) at 20x or 40x magnification. Our selection of based on criteria including Micron per Pixel (MPP), a clear field of view, and high image quality. The TCGA-sourced slide images were randomly divided into training (199 slides), tuning (20 slides), and internal validation (470 slides) groups. The explicit features of cancer or HGD presented on each image grid were annotated by experienced pathologists by delineating the tissues/cells.

***Internal Validation***

The internal validation datasets included 470 FFPE WSIs from 467 patients from the TCGA archive (not overlapping the training data to avoid potential bias). Exclusion criteria refer to WSIs that were not FFPE embedded, not MPP-available, quality-ineligible, or magnification below 20x, and those that had no cancerous tissues or had inconsistent diagnoses from pathologists with synoptic pathology reports. The sensitivity is 99.57%(95%CI: 98.99%,100.00%) on the WSIs.

***Emulated Prospective Study Procedure***

Before study initiation, all four participants were trained to use PathoCruz Viewer via live presentation and operation manuals study, ensuring they acquired competency in using this software freely, as well as the grid-level annotation, which is labeling on the WSI partitioned into 0.5*0.5 mm^2^ grids, was fully understood.

At the medical site, each participant reviewed all WSIs in sequence from the dataset. Each WSI was read twice. At the first reading, the participants were required to click to annotate all grids that had cancer, and when they completed and turned-on AI outputs on that WSI, the initial annotations were stored as the pathologist’s independent diagnosis. During the 2nd review with AI assistance, the participants were allowed to modify their initial annotations on the grids. Modifications were saved when the WSI was closed. Participants completed this work in 90 days, from August 22, 2022, to November 19, 2022.

***Ground Truth for External Validation and the Reader Study***

Ground truth revolves around determining the presence of cancer within each image grid. Each WSI was subdivided in a full mesh style into disjoint grids, each measuring 0.5*0.5 mm^2^. The slide-level ground truth was established by assessing whether any of the grids within a WSI contain cancer.

The ground truth was established separate from the reader study procedure. Two senior pathologists, each with between 5 and 10 years of experience, were tasked with independently annotating grids that exhibited signs of cancer presence on each WSI. Subsequently, a dedicated software program automatically compared their annotations with the outputs generated by the AI system. Any grids that exhibited disagreements between the two pathologists or discrepancies between either pathologist and the AI output were escalated to the chief pathologist for a final decision. It's important to note that the two pathologists conducting the annotations were kept blind to the AI outputs, and they did not participate as WSI readers in the study. Similarly, the chief pathologist remained blinded to both the AI outputs and the annotations made by the two pathologists.
